# Les tubérculomes intracrâniens à Nouakchott, Mauritanie

**DOI:** 10.11604/pamj.2018.30.269.15467

**Published:** 2018-08-09

**Authors:** Sidi Salem-Memou, Samy Dadah, Ahmedou Moctar, Sidelhadj Dah, Ahmed Jeddou, Ahmedsalem Kleib, Sidimohamed Salihy, Outouma Soumare, Mouhamadou Diagana, Najat Boukhrissi

**Affiliations:** 1 Service de Neurochirurgie, Centre Hospitalier National de Nouakchott, Mauritanie

**Keywords:** Tuberculome cérébral, Tuberculose, Mauritanie, Cerebral tuberculoma, tuberculosis, Mauritania

## Abstract

La tuberculose constitue un problème majeur de santé publique dans les pays en voie de développement. Le tuberculome cérébral est une masse de tissu granulomateux tuberculeux ayant été contenu et limité par les défenses immunitaires de l'hôte. Les objectifs poursuivis par ce travail sont de décrire les caractéristiques et d'apprécier le profil évolutif des tuberculomes intracrâniens en Mauritanie. Trente-quatre observations de tuberculome intracrânien ont été colligées de façon rétrospective dans plusieurs hôpitaux de la ville de Nouakchott entre Janvier 2005 et Juin 2017. L'aspect évolutif sous traitement de tous les patients a été analyse. Il s'agissait d'une étude rétrospective ayant porté sur 34 cas de tubérculome intracrânien dont 20 hommes et 14 femmes (sexe ratio de 1,4). L'âge moyen de nos patients est 28.7 ans avec le tiers de la série (12 cas) d'un âge inferieur ou égal a 16 ans. Les signes d'hypertension intracrânienne et les crises convulsives ont dominés la symptomatologie avec respectivement 27 cas (79.41%) et 20 cas (58.82 %). L'intradermoréaction à la tuberculine était positive chez 14 (41.17%). La localisation de la lésion est sus-tensorielle chez 24 patients (70.58%). Sur le plan thérapeutique, tous nos patients avaient bénéficiés d'une une poly chimiothérapie anti tuberculeuse pendant une durée variable supérieure ou égal à 12 mois. 12 de nos patient (35.29%) ont bénéficiés d'un traitement chirurgical. L'évolution a été favorable avec guérison complète sans séquelles dans 23 cas soit 67.64%. Compte tenu de la nature non spécifique de l'imagerie dans le diagnostic du tuberculome intracrânien et de l'absence de la biopsie stéréotaxique dans notre pays où la maladie est endémique, nous recommandons un test thérapeutique de deux mois pour les lésions suspectes.

## Abstract (en)

Tuberculosis is a major public health problem in developing countries. Cerebral tuberculomas is a tuberculous granulation tissue mass restrained and limited by immune defenses of the host. This study aims to describe the characteristics and the evolutionary profile of intracranial tuberculomas in Mauritania. Data of 34 patients with intracranial tuberculoma were collected retrospectively in several hospitals in the city of Nouakchott between January 2005 and June 2017. Evolutionary features of patients under treatment were analyzed. Our retrospective study involved 20 men and 14 women (sex ratio 1.4). The average age of our patients was 28.7 years. Twelve patients were less than or equal to 16 years. Symptomatology was dominated by increased intracranial pressure and seizures in 27 cases (79.41%) and 20 cases (58.82%) respectively. Intradermo tuberculin reaction was positive in 14 (41.17%) cases. Supratentorial lesion was found in 24 patients (70.58%). In all patients, therapeutic approach was based on multidrug chemotherapy for a period greater than or equal to 12 months. Surgical procedure was performed in 12 patients (35.29%). Outcome was favorable with complete healing without sequelae in 23 cases, reflecting a rate of 67.64%. Given the non-specific role of imaging exams in the diagnosis of intracranial tuberculoma and the lack of the stereotactic biopsy in our Country where the disease is endemic, we recommend to perform a two-month therapeutic test in patients with suspicious lesions.

## Introduction

La tuberculose, l'une des maladies infectieuses les plus anciennement connues chez l'homme, est due à des bactéries appartenant au complexe Mycobacterium Tuberculosis. En ce début de XXI^ème^siècle, la tuberculose sévit encore de manière endémique dans nos régions où elle pose un véritable problème de santé publique de par sa recrudescence liée surtout à un bas niveau économique et à la pandémie du SIDA [1]. La tuberculose cérébrale reste parmi les plus graves des formes extra pulmonaires. L'atteinte cérébro-méningée, se fait surtout par voie hématogène à partir d'un foyer primaire le plus souvent pulmonaire. Le tuberculome est une masse de tissu granulomateux tuberculeux ayant été contenu et limité par les défenses immunitaires de l'hôte. Il se présente comme une lésion expansive intracrânienne. Le diagnostic est basé sur un faisceau d'arguments anamnestiques, clinico-biologiques et radiologiques. La confirmation reste histologique. Sa prise en charge est pluridisciplinaire et repose sur la chimiothérapie antituberculeuse. Les objectifs poursuivis par ce travail sont de décrire les caractéristiques cliniques, paracliniques, thérapeutiques, et d'apprécier le profil évolutif sous traitement des tuberculomes intracrâniens en milieu hospitalier à Nouakchott (Mauritanie).

## Méthodes

Il s'agit d'une étude rétrospective, menée dans plusieurs hôpitaux de la ville de Nouakchott entre Janvier 2005 et Juin 2017. Etaient inclus les patients pris en charge durant cette période et chez qui le diagnostic de tuberculome intracrânien a été retenu. Le diagnostic de tuberculose était soit confirmé par des résultats bactériologiques ou anatomopathologiques soit présomptif retenu devant un faisceau d'arguments épidémiologiques, cliniques, biologiques, morphologiques et évolutifs sous traitements spécifiques. Les patients ayant une méningite tuberculeuse isolée, ou ayant des dossiers non exploitables ont été exclus. Tous les dossiers retenus ont été étudiés en précisant les aspects épidémiologiques, les caractéristiques cliniques, les données de l'imagerie, de la biologie ainsi que les aspects évolutifs sous traitement.

## Résultats

Trente-quatre observations de tuberculome intracrânien ont été colligées dont 20 hommes et 14 femmes (sexe ratio de 1,4). L'âge moyen de nos patients était 28.7 ans avec des extrêmes allant de 09 à 60 ans. Les enfants représentaient le 1/3 de la série (12 cas) avec un âge compris entre 09 et 16 ans. Tous nos patients avaient un bas niveau socio-économique et 75% d'entre eux résidaient en zone rurale. La vaccination antérieure par le BCG était retrouvée dans 20 cas (58.82 %). Six de nos patients (17.64%) avaient des antécédents personnels de tuberculose pulmonaire avérée. La notion de contage tuberculeux était retrouvée dans 12 cas (35.29 %) et 4 cas (11.76 %) de déficit immunitaire ont été répertoriés. Sur le plan clinique (Tableau 1), la durée d'évolution des symptômes variait de 3 à 16 mois avec une moyenne de 8 mois. Les signes d'hypertension intracrânienne et les crises convulsives ont dominés le tableau chez respectivement 27 cas (79.41%) et 20 cas (58.82 %). La fièvre au long court était présente chez 12 cas (35.29%) et l'altération de l'état général avec amaigrissement, asthénie et anorexie dans seulement 3 cas (8.82%). Douze patients (35.29%) étaient admis avec des troubles de la conscience, allant de la somnolence au coma. Des signes de localisation à type de déficit moteur étaient observés chez 13 cas (38.23%). Un cas (2.9%) avait une cécité bilatérale. Au plan biologique, une intradermoréaction à la tuberculine était positive (10-18mm) chez 14 cas (41.17%) des 20 cas chez lesquels elle était réalisée. Une hyperalbuminorrachie d'en moyenne 3g/litre était observée chez 5 cas. La recherche de BK dans les expectorations était effectuée chez tous nos patients, et positive chez 2 cas. La recherche de mycobactérie sur l'examen direct du LCS était négative dans les 7 cas qui ont eu un prélèvement. Il n'y a pas eu de culture ni de recherche par méthode de polymérase chain reaction (PCR) au niveau du LCS. La CRP était positive dans 15 cas (44.11%) et la vitesse de sédimentation dans 20 cas (58.82%).

**Tableau 1:** Répartition des symptômes et signes cliniques chez 34 patients porteurs de tuberculome intracranien, en fonction de l’âge dans les services de neurochirurgie à Nouakchott, Mauritanie de 2005 à 2017

Symptômes et signes cliniques	Enfant Effectif(n)	Adulte Effectif (n)	Total (%)
Céphalées	8	21	29 (85.29)
Vomissements	8	19	27(79.41)
Convulsions	3	17	20 (58.82)
Œdème papillaire	5	5	10(29.41)
Atrophie optique	1	1	2(5.88)
Atteinte de nerfs crâniens	2	5	7(20.58)
BAV	3	5	8(23.52)
Ataxie cérébelleuse	1	4	5(14.70)
Hémiparésie	3	10	13(38.23)
Troubles de la conscience	4	8	12(35.29)

**Figure 1:**
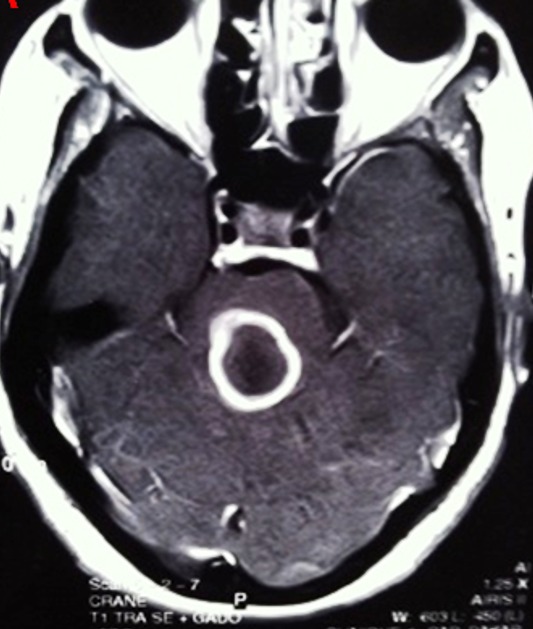
IRM cérébrale T1 après injection de produit de contraste montrant un abcès tuberculeux du tronc cérébral

**Figure 2:**
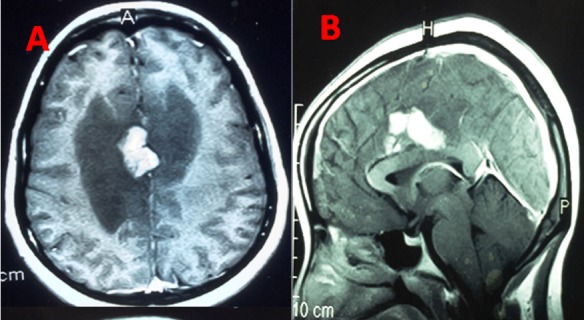
IRM cérébrale T1 après injection de produit de contraste montrant une lésion pariétale interne prenant fortement le contraste avec œdème périlésionnel

Une tomodensitométrie cérébrale sans et avec injection de produit de contraste était réalisée chez tous nos cas. La localisation de la lésion était sus-tensorielle chez 24 cas (70.58%). La lésion était unique dans 27 cas (79.41%), multiple chez 7cas (20.58%) et bilatérale chez 3 cas (8.82%). La localisation dans la fosse cérébrale postérieure a été retrouvée dans 10 cas, il s'agissait de 2 tuberculomes du tronc cérébral (Figure 1, Figure 2) et de 8 cérébelleux. Une hydrocéphalie était associée chez 5 cas (14.7%) (Porteurs de lésion sous-tentorielle). Une réaction méningée était trouvée chez 4 cas (11.76 %). L'IRM cérébrale n'a été réalisée que chez 8 cas. La radiographie du thorax était faite systématiquement et n'était pathologique que dans 4 cas (11.76 %). Le diagnostic a été confirmé à l'examen histologique chez 9 cas dans notre série. Au plan thérapeutique, tous nos patients ont bénéficiés d'un traitement avec une poly chimiothérapie anti tuberculeuse pendant une durée variable supérieure ou égal à 12 mois. Douze (12) de nos patient (35.29%) avaient bénéficié d'un traitement chirurgical à type de chirurgie d'exérèse complète chez 5 cas et de biopsie cérébrale par trépano-ponction à main levée chez 7 cas (la biopsie stéréotaxique étant non disponible). L'hydrocéphalie a été traitée par valve de dérivation ventriculo-péritonéale chez 4 cas et par ventriculocisternostomie endoscopique chez un cas. Des tomodensitométries de contrôle étaient réalisées chez tous les patients à la fin du traitement. Ils révélaient une complète résorption des lésions chez 19 cas (82%), alors que chez 5 cas il restait une prise de contraste et chez 3 cas une petite zone iso-dense sans œdème péri-lésionnel. L'évolution a été favorable avec guérison complète sans séquelles chez 23 cas (67.64%). Sept patients (20.5%) avaient des séquelles mineures et 2 cas (5.8%) des séquelles majeures. Deux patients étaient décédés en cours de traitement.

## Discussion

La tuberculose du système nerveux central (SNC) représente 1% de l'ensemble de la tuberculose. Elle comporte la méningite tuberculeuse, les tuberculomes cérébraux, l'encéphalite tuberculeuse et les accidents vasculaires cérébraux au cours de l'atteinte tuberculeuse du SNC. Elle est la forme la plus sévère des tuberculoses extra pulmonaires avec un taux élevé de mortalité et de séquelle nerveux résiduel [2]. Le tuberculome intracrânien est l'une des fromes de tuberculoses du SNC, il s'agit d'une masse de tissu granulomateux tuberculeux ayant été contenue et limitée par les défenses immunitaires de l'hôte. Il se présente comme une lésion expansive intracrânienne [3, 4]. Les tuberculomes cérébraux sont communs dans les pays en voie de développement où ils restent une localisation importante de la maladie tuberculeuse avec une incidence assez élevée, ils peuvent représenter 5 à 40 % des processus expansifs intracrâniens [5, 6]. La difficulté d'accès aux soins, les conditions d'hygiène précaires, la promiscuité et la malnutrition sont des facteurs de risque favorisant la survenue des tuberculomes d'où la forte endémie tuberculeuse dans les pays en voie de développement [7]. Dans notre étude la quasi-totalité des patients proviennent de zones défavorisées notamment rurales. Dans les pays développés, le tuberculome reste une lésion rare bien que son incidence soit en augmentation, en rapport avec la population immigrée de pays où la tuberculose est endémique [8]. Le tuberculome intracrânien résulte d'une diffusion hématogène à partir d'un foyer primitif, il débute sous forme d'un conglomérat de microgranulomes dans une zone d'encéphalite et confluent par la suite pour former un tuberculome mature non caséifié [9]. Il peut se voir a tous les âges de la vie, aussi bien chez l'enfant que chez l´adulte [8, 10-14], a l´exception des tout-petits. Dans les pays à forte endémie, les tuberculomes sont particulièrement fréquents chez les enfants ; néanmoins cette pathologie concerne exceptionnellement l´enfant de moins de 2 ans [15]. Les tuberculomes peuvent siéger n'importe où dans le cerveau. Classiquement, la localisation a plutôt tendance à être sous-tentorielle chez l'enfant, sus-tentorielle chez l'adulte [16, 17]. Les localisations les plus fréquentes sont : les hémisphères cérébraux, le cervelet, le tronc cérébral et le chiasma optique. D'autres localisations plus rares à savoir : tubercules quadrijumeaux, corps calleux, amygdales cérébelleuses, plexus choroïdes, troisième ventricule, espace sous-dural, cavités mastoïdiennes ou du sinus caverneux [18, 19]. II faut souligner la possibilité de double localisation supra et sous-tentorielle [14].

Les signes neurologiques révélateurs sont ceux d'un processus expansif intracrânien et sont directement en rapport avec la topographie de la lésion. Il peut ainsi s'agir de céphalées, d'une hypertension intracrânienne, de déficits neurologiques focaux, de crises d'épilepsie ou encore d'une confusion [20-23] Les éléments d'orientation diagnostique tels qu'une tuberculose extra crânienne évolutive, des antécédents de tuberculose, de contage tuberculeux et les signes généraux tels que fièvre et amaigrissement peuvent manquer [20, 24]. Cependant, les tuberculomes cérébraux sont habituellement associés à une autre localisation tuberculeuse, généralement pulmonaire, qui peut être occulte mais qui est responsable d'une diffusion hématogène vers le parenchyme cérébral [22, 23]. Rarement, les tuberculomes cérébraux s'associent à une méningite tuberculeuse [21] qui peut être le foyer d'origine mais qui peut aussi être secondaire à la rupture d'un tuberculome dans l'espace sous-arachnoïdien [25]. Les éléments de la biologie peuvent donner une orientation diagnostique. Le test tuberculinique peut être positif et la réaction a tendance à être plus forte chez les patients présentant une atteinte extra-crânienne. Néanmoins, Il est reconnu qu´un test de tuberculine positif a une valeur diagnostic limitée dans les communautés où la tuberculose est endémique, En raison de l´exposition fréquente aux mycobactéries. Une intradermo-réaction importante à la tuberculine (supérieure à 15mm) traduit une infection tuberculeuse active même en zone endémique [26]. L'introduction de l'imagerie médicale dans notre pays (scanner puis IRM plus tard) a sensiblement amélioré le diagnostic de présomption des tuberculomes avec un nombre de cas plus important depuis l'avènement de l'IRM. Le tuberculome caséifié est la forme la plus classique. Il apparaît en TDM isodense, discrètement hyperdense par rapport au parenchyme cérébral. Après injection, il ne prend pas le contraste à l'exception de sa périphérie, la partie granulomateuse du tuberculome, ce qui lui donne un aspect en couronne, très évocateur, sans être pour autant spécifique. Quelquefois, on peut observer des calcifications dans la partie centrale [27]. L'aspect IRM du tuberculome est variable en fonction de son stade evolutif : les tuberculomes non caséifiés paraissent en hyposignal T1 et hypersignal T2 par rapport au parenchyme cérébral et se rehaussent de façon intense et homogène par le produit de contraste. Les lésions caséifiées a centre solide paraissent en hypo ou isosignal T1 et en hypo ou isosignal T2 et s'associent souvent a de l'œdème perilesionnel. Les tuberculomes caséifiés à centre nécrotique paraissent en hyposignal T1 et hypersignal T2 et se rehaussent en périphérie par le produit de contraste. Les séquences d'imagerie par diffusion et par spectroscopie sont actuellement très utiles pour le diagnostic précoce et non invasif des tuberculomes cérébraux surtout chez les patients sans signes systémiques de tuberculose. Elles permettent aussi une caractérisation spécifique des tuberculomes, par rapport aux autres lésions infectieuses et aux lésions tumorales primitives et secondaires [28, 29].

**Figure 3:**
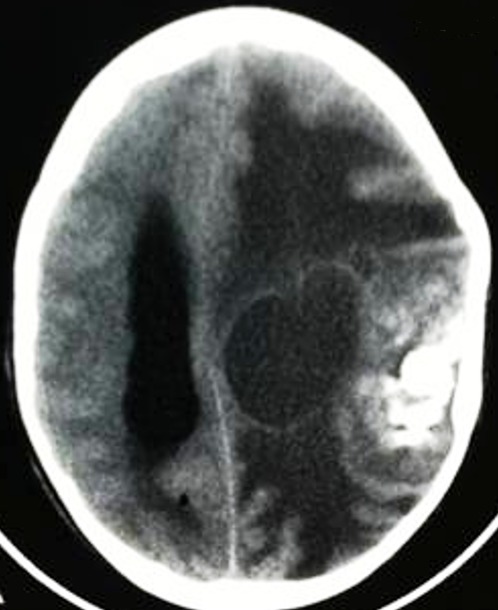
Scanner cérébral après injection du produit de contraste montrant un tuberculome pariétal à double composante, charnue calcifiée et kystique

**Figure 4:**
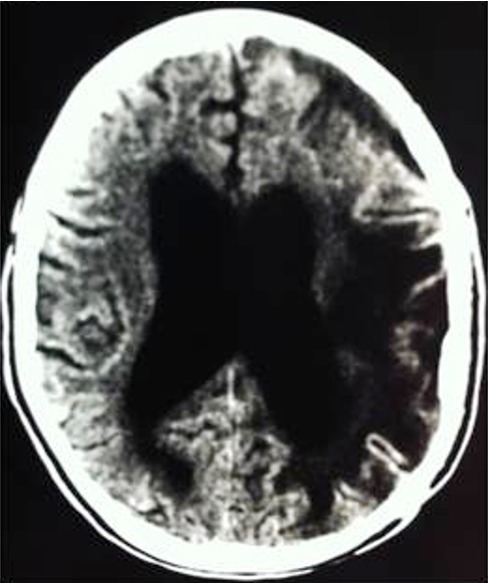
Scanner cérébral de contrôle du patient de la figure 3, après chirurgie et traitement anti-bacillairede 9 mois montrant l’absence de lésion

**Figure 5:**
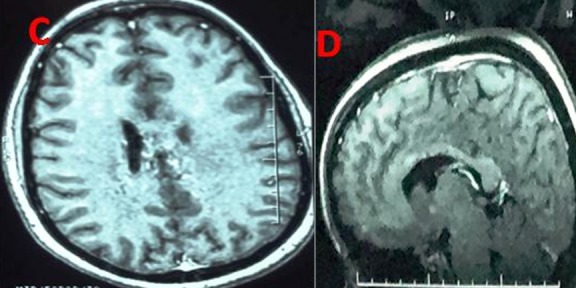
IRM cérébrale T1 de contrôle du patient de la figure 2, après 12 mois de traitement antituberculeux montrant l’effacement complet de la lésion

Le diagnostic différentiel surtout devant des lésions multiples, se pose avec des localisations secondaires. Celles-ci sont habituellement plus œdématogénes que les tuberculomes. Chez les patients séropositifs pour le VIH, il faut évoquer les abcès toxoplasmiques et les lymphomes. Sous l'effet du traitement médical, l'involution du tuberculome se fait lentement sur plusieurs mois, et la résolution définitive n'est souvent obtenue que très tardivement. La régression en taille est lente dans le premier mois (7,3%) puis rapide de 15 à 20 % par mois [3]. L'œdème disparaît progressivement en 6 mois. Dans notre étude; l'évolution sous traitement a été remarquablement favorable, et dans la majorité des cas (80%) les lésions étaient complètement effacées dans les 18 mois (Figure 3, Figure 4, Figure 5). L'efficacité du traitement antituberculeux a rendu guérissable cette pathologie potentiellement mortelle et a amélioré de façon considérable son pronostic notamment en réduisant le nombre d'intervention chirurgicale pour tuberculome intracrânien. Toutefois, la chirurgie se justifie en cas d'hypertension intracrânienne menaçante, altération de l´acuité visuelle, hydrocéphalie sur tuberculome de la fosse postérieure, échec du traitement antituberculeux, l'augmentation paradoxale de la taille sous traitement médical et devant un doute diagnostique [30,31]. Dans notre contexte de zone d'endémie, la preuve systémique associée à des images radiologiques évocatrices constitue une indication à la mise en route du traitement antituberculeux. De même qu'un aspect radiologique très évocateur à lui seul peut autoriser la mise en route de la chimiothérapie antituberculeuse test surtout pour les localisations uniques ou multiples en zone fonctionnelles ou profondes. Compte tenu de la nature non spécifique de l'imagerie la tomodensitométrie et de l'imagerie par résonance magnétique dans le diagnostic du tuberculome intracrânien et de l´absence de la biopsie stéréotaxique dans notre pays où la maladie est endémique, nous recommandons un test thérapeutique de deux mois pour les lésions suspectes

## Conclusion

La tuberculose cérébrale est une forme sévère de la tuberculose neuromeningée. Il faut y penser devant toute image suspecte. La mise en route d'un traitement médical de preuve par anti-tuberculeux d'au moins 2 mois semble être l'attitude diagnostic et thérapeutique la plus logique dans notre contexte endémique.

### Etat des connaissances actuelles sur le sujet

- Le tuberculome intracrânien se comporte comme un processus expansif intracrânien ;
- La tuberculose sévit encore de manière endémique en Afrique ;
- Le traitement anti-bacillaire est très efficace sur tous les types de la tuberculose.

### Contribution de notre étude à la connaissance

- Notre étude confirme la pertinence du traitement d'épreuve dans les pays endémique;
- Notre étude apporte l'expérience mauritanienne dans la prise en charge des tuberculomes intracrâniens.

## Conflits d’intérêts

Les auteurs ne déclarent aucun conflit d'intérêts.
